# Frequency of *COL4A3/COL4A4* Mutations amongst Families Segregating Glomerular Microscopic Hematuria and Evidence for Activation of the Unfolded Protein Response. Focal and Segmental Glomerulosclerosis Is a Frequent Development during Ageing

**DOI:** 10.1371/journal.pone.0115015

**Published:** 2014-12-16

**Authors:** Louiza Papazachariou, Panayiota Demosthenous, Myrtani Pieri, Gregory Papagregoriou, Isavella Savva, Christoforos Stavrou, Michael Zavros, Yiannis Athanasiou, Kyriakos Ioannou, Charalambos Patsias, Alexia Panagides, Costas Potamitis, Kyproula Demetriou, Marios Prikis, Michael Hadjigavriel, Maria Kkolou, Panayiota Loukaidou, Androulla Pastelli, Aristos Michael, Akis Lazarou, Maria Arsali, Loukas Damianou, Ioanna Goutziamani, Andreas Soloukides, Lakis Yioukas, Avraam Elia, Ioanna Zouvani, Polycarpos Polycarpou, Alkis Pierides, Konstantinos Voskarides, Constantinos Deltas

**Affiliations:** 1 Molecular Medicine Research Center and Laboratory of Molecular and Medical Genetics, University of Cyprus, Nicosia, Cyprus; 2 Department of Nephrology, Evangelismos Hospital, Pafos, Cyprus; 3 Department of Nephrology, Nicosia General Hospital, Nicosia, Cyprus; 4 Department of Nephrology, Larnaca General Hospital, Larnaca, Cyprus; 5 Department of Nephrology, Limassol General Hospital, Limassol, Cyprus; 6 Department of Nephrology, Paphos General Hospital, Paphos, Cyprus; 7 Department of Pediatrics, Archbishop Makarios III Hospital, Nicosia, Cyprus; 8 Department of Histopathology, Nicosia General Hospital, Nicosia, Cyprus; 9 Department of Nephrology, Apollonion Private Hospital, Nicosia, Cyprus; 10 Department of Nephrology, Hippocrateon Hospital, Nicosia, Cyprus; University of Delaware, United States of America

## Abstract

Familial glomerular hematuria(s) comprise a genetically heterogeneous group of conditions which include Alport Syndrome (AS) and thin basement membrane nephropathy (TBMN). Here we investigated 57 Greek-Cypriot families presenting glomerular microscopic hematuria (GMH), with or without proteinuria or chronic kidney function decline, but excluded classical AS. We specifically searched the *COL4A3/A4* genes and identified 8 heterozygous mutations in 16 families (28,1%). Eight non-related families featured the founder mutation *COL4A3-*p.(G1334E). Renal biopsies from 8 patients showed TBMN and focal segmental glomerulosclerosis (FSGS). Ten patients (11.5%) reached end-stage kidney disease (ESKD) at ages ranging from 37-69-yo (mean 50,1-yo). Next generation sequencing of the patients who progressed to ESKD failed to reveal a second mutation in any of the *COL4A3/A4/A5* genes, supporting that true heterozygosity for *COL4A3/A4* mutations predisposes to CRF/ESKD. Although this could be viewed as a milder and late-onset form of autosomal dominant AS, we had no evidence of ultrastructural features or extrarenal manifestations that would justify this diagnosis. Functional studies in cultured podocytes transfected with wild type or mutant *COL4A3* chains showed retention of mutant collagens and differential activation of the unfolded protein response (UPR) cascade. This signifies the potential role of the UPR cascade in modulating the final phenotype in patients with collagen IV nephropathies.

## Introduction

During the last three decades, progress in molecular genetics has allowed better investigation and understanding of patients with familial microscopic hematuria (FMH) of glomerular origin. Six genes (*COL4A3, COL4A4, COL4A5, CFHR5, MYH9, FN1*) have so far been identified that when mutated, cause this disorder [1]. Mutations in *COL4A3/A4/A5*, coding for the alpha chains of the trimeric collagen IV, the most important structural component of the glomerular basement membrane (GBM) [2], [3] appear to be responsible for the two most frequent causes of FMH. These are thin basement membrane nephropathy (TBMN) and Alport Syndrome (AS) [4], [5].

MH of glomerular origin is the earliest presenting sign of AS and TBMN but their long term clinical outcome is not always predictable, especially in patients who are heterozygous for *COL4A3/A4* mutations. In many families carrying such mutations, some members continue to exhibit pure and isolated MH for the rest of their lives, while others develop proteinuria later on in life, usually with hypertension and a variable gradual progression to CRF, up to ESKD [6]–[8]. This phenotypic heterogeneity raises several aetiologic questions and has great clinical relevance.

In the present study, we focused only on the *COL4A3* and *COL4A4* genes. These genes are composed of an N-terminal 7S domain, a triple-helical collagenous domain with the characteristic repetitive Gly-X-Y motif, and a C-terminal noncollagenous globular domain (NC1 domain). The NC1 domain is crucial for directing chain recognition and assembly for forming the collagen IV heterotrimers. The sequence and structure embedded in the NC1 domain ensures that the only three types of trimers that are biochemically permissible are: α1α1α2, α3α4α5, α5α5α6. This selection process takes place in the endoplasmic reticulum, where the three α-chains fold to form the protomer, which subsequently undergoes a series of post-translational modifications before secretion to the GBM [9]. GBMs are very thick structures (average width: female 326 nm, male 373 nm) that play a crucial role in establishing and maintaining an effective and properly functioning glomerular filtration barrier (GFB) [10]. This GFB consists of three layers, two of which, the innermost vascular endothelium and the outside podocytes are cellular, while the third one, the GBM is acellular and lies between the other two. It is now recognized that the mature type IV collagen network, α3α4α5, originates solely in the podocytes [11].

Following the description of the X-linked form of AS (XLAS) in the early 1990’s [12], [13] the rarer ARAS was also described and explained by homozygous or compound heterozygous mutations in either the *COL4A3* or *COL4A4* genes [14], [15]. In 1996 Lemmink et al recognized that a common form of FMH of glomerular origin associated with TBMN and usually normal kidney function, was the result of inheritance of heterozygous mutations in the same *COL4A3/A4* genes [16]. Follow up studies since then have suggested that heterozygous *COL4A3/A4* mutations may explain about 40% of families with FMH and TBMN and our data substantiate these figures. No additional genes have yet been cloned, associated with TBMN. Some authors also recognize an autosomal dominant form of AS, caused by heterozygous *COL4A3/A4* mutations and Alport-like ultrastructural histology [7], [17].

During our initial work on large and mostly symptomatic Greek-Cypriot families with FMH we had identified three founder *COL4A3/A4* mutations in patients who manifested the dual diagnosis of focal and segmental glomerulosclerosis in the presence of TBMN. Mutation *COL4A3*-p.(G1334E) was particularly common and accounts for a significant subset of patients with FMH due to TBMN, as a strong founder effect [6]. In the present study we aimed to find the frequency of *COL4A3/A4* mutations in a much larger Cypriot population with FMH that did not necessarily show additional renal findings. In a total of 57 consecutive families that were referred to our center during the period of 2009 to July 2011, eight heterozygous mutations were detected in 87 patients of 16 families (28,1%). Notably, among patients carrying heterozygous mutations, 51,6% of patients older than 51 years progressed to CRF. Equally important is that NGS DNA analysis of ten patients who progressed to ESKD failed to detect a second mutation in either of *COLA3/A4/A5* genes. Remarkably, in 14 of 41 families that we did not find *COL4A3/A4* mutations, there were 54 patients who had solely manifested isolated GMH. Presumably these families and patients have inherited mutations in other, yet unknown, less deleterious genes.

In addition, we examined in cell culture experiments the effect some of these mutations have on the induction of ER stress and the UPR pathway [18]. In a recent study we had shown that over-expression of wild type (WT) or mutant collagen chains in human podocytes differentially activate the UPR pathway, thereby implicating this process in a cellular model of disease [19]. Activation of the UPR signaling cascade signifies the situation where proteins are synthesized and accumulated in excess or are misfolded in the ER, thereby leading the cell towards an adaptive or a maladaptive pathway, depending on the duration and severity of the triggering event [18], [20].

## Materials and Methods

### Patients

Patients belonging to 57 Greek-Cypriot families with FMH of glomerular origin that were consecutively referred to our center since our previous relevant publications [21] and until July 2011, were investigated clinically and molecularly. Altogether there were 304 affected members, currently alive or deceased. Inclusion criteria required that all families had a minimum of three affected subjects with isolated GMH or GMH plus proteinuria and CRF. IgA nephropathy patients were also excluded. Other non-glomerular causes of hematuria were excluded. Renal biopsies were available for patients of 21/57 (36.8%) families, since biopsies were not routinely performed in the presence of isolated GMH or GMH and low grade proteinuria (14 families). Among the 16 families with confirmed mutations, there was a biopsy proven histological diagnosis of TBMN in the presence of FSGS, in only five patients. In a sixth patient the biopsy showed FSGS but no EM studies were available. None of these five biopsies had GBM splitting or other ultrastructural findings suggestive of classical AS. In most cases the molecular testing preceded the biopsy and consequently a positive molecular finding rendered the invasive biopsy procedure even more unlikely. CRF was defined as an elevated serum creatinine >1.5 mg/dL.

This research project was approved by the Cyprus National Bioethics Committee and all subjects gave their informed signed consent.

### Mutation screening and DNA sequencing

Molecular testing by DNA linkage analysis, mutation screening and/or direct DNA sequencing was our approach for investigation, performed as previously described [6]. A renal biopsy was avoided unless it was deemed necessary on clinical grounds. DNA from peripheral blood leucocytes was isolated by a salting out procedure [22] or by using the Qiagen Kit (Qiagen, Hilden, Germany). All 100 exons of *COL4A3/A4* genes were screened for mutations using the SURVEYOR endonuclease (Transgenomic, Cheshire, UK), which cleaves double-stranded DNA at positions of mismatches, as previously described [23], [24]. A slight modification was that incubation occurred at 42°C for 17 min. When cleavage was evident, DNA re-sequencing was performed using the ABI BigDye Terminator v1.1 Cycle Sequencing Kit and the ABI PRISM 3130*xl* genetic analyzer. The oligonucleotide sequences were adopted from a previous publication [6]. After sequencing, restriction enzymes were used for examination of additional family members and healthy controls (Tables 1, 2).

**Table 1:** Τable 1. Information on mutations and reagents used for their identification.

Mutation	Forwardprimer	Reverseprimer	PCRsize (bp)	Tm(°C)	Restrictionenzyme	Cleavageproducts (bp)
COL4A3-p.G1334E	GCACACTTCTA GTATTTGTCCTTAGAGTC	GAAGTTGTAT CAGCTGTTTCCAAAG	477	65	*Hpy188III*	143+334(mutant)
COL4A3-p.G871C	GTTAGTAGGGG AAAGCATTTGTGG	CTATGTACAG TTGACAGAGCCACCT	298	64	*HpyCH4III*	177+121(mutant)
COL4A3c.2621-2622delGAinsT	GTTAGTAGGGG AAAGCATTTGTGG	CTATGTACAG TTGACAGAGCCACCT	298	64	*EcoRV*	194+107(normal)
COL4A3-p.G1077D	GTGCTGGCAGA TAGCAGATACTAA	GATTTCAGGA GGGCTATACTCTGA	323	63	*HphI*	162+161(normal)
COL4A3-p.G484R	GTTCTTTCTGAG GACTCAATGTAGCTT	CTTCCAGTGT ATTGACCCTTTTGT	213	60	*Sau96I*	100+113(normal)
COL4A4-c.3854delG	CAGAACCAGCC ACTCCTCTGCC GCTATTGGGAAGTGG	CTTTCCACGA GGACCTGGAG GAGAGATTCCTGGGCTGC	214	62	*SfcI*	175+39(normal)
COL4A4-p.G143V	CTACGTAGCCT TTTGGGGTAAAG	CCAGGCACAC TTGTATTAACTCTG	375	58	*BsmaI*	213+162(mutant)
COL4A4g.227958889-227958838del52	CGATAACCTAA GCAAGTGTGTACC	GGATGTGAAA GTCCAACTTCAG	428	65	NA	NA
COL4A4-p.G208D	CAAAGCTGCTG TTGAAAATGTC	GATAATGGTG GGTTTTCACTGATTC	360	60	*EcoO109I*	182+178(mutant)
**Variant of** **unknown** **significance**						
COL4A4-p.G1433D	GGCATACGGTA TAAGCACGGTAA	GAAAGCCACT TGAGAGATCAGAG	279	64	NA	NA

If no restriction enzyme is given, detection was performed by direct Sanger DNA sequencing.

**Table 2:** Mutations detected in *COL4A3* and *COL4A4* genes.

Family	Gene	Exon	Change inDNA	Change inamino-acid	Controlstested	Reference	SNPs3D	SIFT	Grantham	Polyphen-2
CY-5371,CY-5374,CY-5375,CY-5376,CY-5352,CY-5403,CY-5419,CY-5442	COL4A3	45	c.4001GGA>GAA	p.G1334E	ND	[6], [55]	ND	ND	98	1.000
CY-5346,CY-53401	COL4A3	32	c.2611 GGT>TGT	p.G871C	ND	[6]	ND	ND	159	1.000
CY-5461	COL4A3	23	c.1450GGG>AGG	p.G484R	0/105CY* +	Thisstudy	−2.13	0.00	125	1.000
CY-4204	COL4A3	32	c.2621- 2622delGAinsT	p.Gly874fs8*	0/100CY*	[55]	NA	NA	NA	NA
CY-4204,CY-5322	COL4A3	38	c.3229GGT>GAT	p.G1077D	0/110CY*	Thisstudy	−4.04	0.00	94	1.000
CY-5321	COL4A4		c.3854delG	p.Ser1217fs1287*	ND	[6], [56]	NA	NA	NA	NA
CY-5430	COL4A4	7	c.428GGC>GTC	p.G143V	0/100CY* +	Thisstudy	−3.71	0.00	109	1.000
CY-5324	COL4A4	10	c.623GGT>GAT	p.G208D	0/100CY* +	Thisstudy	−2.52	0.00	NA	1.000
RO-5470(Romanian)	COL4A4	20	g.227958889-227958838del52		ND	Thisstudy	NA	NA	NA	NA
CY-5328	COL4A4	45	c.4298GGT>GAT	p.G1433D	6/120CY**	Thisstudy	−3.41	0.00	NA	1.000

*these mutations tested negative in an additional collection of 52 patients with chronic kidney disease.

**this mutation was detected only in a single patient during screening of 153 patient samples. It was subsequently detected in six of 120 Cypriot controls. It is a suspect founder mutation and is under further investigation.

+ these mutations tested negative in an additional collection of 40 patients with glomerulonephritis of unknown aetiology CY, Cypriot samples; RO, Romanian sample; ND: Not Done; NA: Not applicable.

The pathogenicity of DNA variants was assessed *in silico* with the use of various softwares, as follows: *SIFT* takes a query sequence and uses multiple alignment information to predict tolerated and deleterious substitutions for every position of the query sequence. Positions with normalized probabilities less than 0.05 are predicted to be deleterious; those greater than or equal to 0.05 are predicted to be tolerated [25]. *SNPs3D* prediction is based on two models, the first taking into account that many disease SNPs decrease protein stability and the second based on analysis of homology sequence families related to human proteins. A positive score indicates a variant classified as non-deleterious, and a negative score indicates a deleterious variant [26]. The *Grantham* score evaluates the effect of an amino acid substitution on a protein sequence, based on the natural physicochemical properties of amino acids. The minimum score is 0 and the maximum is 215. Higher scores correspond to higher differences in chemical properties, thus increase the probability of a substitution being damaging to protein function [27]. *Polyphen 2* is an online tool widely used to predict the putative impact on protein function caused by an amino acid residue change and takes into account a variety of attributes such as protein structure, sequence nature and conservation. *Polyphen 2* scores range from 0 to 1 and higher values indicate an increased probability of the amino acid substitution being damaging [28].

### Next Generation Sequencing (NGS)

A custom Ampliseq panel was designed using the Ion Ampliseq Designer V.3.0 (LifeTechnologies, Carlsbad, CA, USA) that included the *COL4A3*, *COL4A4* and *COL4A5* genes. This design was comprised of 260 primer pairs that amplify exonic sequences with ±10 bp padding (59.23 kb per patient in total). Coverage for all exons was 99.09%, with 9 bp being left out from *COL4A4*, 107 bp from *COL4A3* and 53 bp from *COL4A5*. All missing regions were screened in all samples with Sanger sequencing. Amplicon sizes ranged from 125–175 bp. Library was built by amplifying 10 ng of genomic DNA, using the Ion Ampliseq Library Kit v2.0 (LifeTechnologies). Each sample was barcoded for multiplexing. Ampliseq library quantification and enrichment validation was performed using the Taqman Ion Library Quantitation Kit (LifeTechnologies) on a ViiA-7 Real Time PCR machine (Applied Biosystems, Foster City, CA, USA). Amplified libraries were pooled and loaded onto the Ion OneTouch 2 system (Life Technologies) for emulsion PCR and enrichment of Ion Sphere Particles (ISPs) followed, using the OneTouch ES system (Life Technologies). Enriched ISPs were then loaded on an Ion 316 chip and sequenced on the Ion Torrent PGM sequencer (Life Technologies). Sequencing generated the corresponding BED files for each sample, which were then submitted for analysis. The mean coverage depth of all amplicons achieved by NGS for all samples was 270x. Amplicons with depth of coverage of less than 20x were additionally examined by Sanger sequencing. Base calling, sequence alignment against the hg19 reference genome and variant calling and annotation were performed by the Torrent Suite V4.0.2 Variant Caller V4.0 (Life Technologies) and the Ion Reporter Software V4.0 (Life Technologies) using default or optimized parameters. Further, results were visualized after being analyzed in the Broad’s Integrative Genomics Viewer (IGV). All variants were annotated by a number of available databases, including the NCBI dbSNP138 and ClinVar [29], while MAF scores were obtained by the 1000 Genomes project. The potential effect of each newly discovered variant was assessed by the PolyPhen2 (http://genetics.bwh.harvard.edu/pph2/), SIFT (http://sift.jcvi.org/) and Grantham algorithms. False positives were filtered out after examining data for strand bias (MNP Strand Bias value set at 0.95). Each variant was assessed using standard dye-terminator sequencing, on the ABI3130*xl* (Applied Biosystems) with BigDye v1.1 chemistry and relevant primers. Results were visualized with Broad Institute’s Integrative Genomics Viewer (IGV). BAM files were submitted to the European Nucleotide Archive (ENA) under the accession number PRJEB7473.

### Plasmid vectors

A plasmid vector containing the full-length human *COL4A3* cDNA subcloned into the pCMV6-AC-HA was supplied from Origene (Rockville, Maryland, USA). The *COL4A3*-p.(G484R), *COL4A3*-p.(G871C) and *COL4A3*-p.(A587G) point mutations were introduced in the WT *COL4A3* by PCR-based site-directed mutagenesis (QuickChange Site-Directed Mutagenesis, Stratagene, La Jolla, CA). The *COL4A3*-p.(G1334E) mutation was used as a positive control for activation of UPR markers [19]. DNA sequencing and restriction digestion were performed in order to confirm mutants. See Table 3 for primers.

**Table 3:** Details of mutagenic primers used in this work for introducing mutations in cDNA constructs that were transiently transfected into cultured podocytes AB8/13.

	Oligonucleotidename	Sequence	Length
1	COL4A3_G484R_F	CCCTTATATCCCA**A**GGCCTCCCGGTCTCCC	30
2	COL4A3_G484R_R	GGGAGACCGGGAGGCC**T**TGGGATATAAGGG	30
3	COL4A3_G871C_F	GGTGAAATGGGACCACTG**T**GTCAAAGAGGATATCCAGG	38
4	COL4A3_G871C_R	CCTGGATATCCTCTTTGAC**A**CAGTGGTCCCATTTCACC	38
5	COL4A3_A587G_F	CCTAAAGGCGAACTGG**G**TCTGAGTGGTGAGAAAG	34
6	COL4A3_A587G_R	CTTTCTCACCACTCAGA**C**CCAGTTCGCCTTTAGG	34
7	COL4A3_G1334E_F	CCATTGGACCTCCAG**A**ACCAATTGGGCCAAAAGG	34
8	COL4A3_G1334E_R	CCTTTTGGCCCAATTGGT**T**CTGGAGGTCCAATGG	34

### Cell culture and transfections

The AB8/13 human podocyte cell line was kindly provided by MA Saleem and cultured as previously described [30]. At 70% confluence, cells were transiently transfected with the vectors containing the collagen cDNAs, WT or mutant, using lipofectamine 2000 (Invitrogen, California, USA) and according to manufacturer’s instructions. Cellular lysates were collected for experiments 48 hours after transfection. In all experiments the empty pCMV6-AC-HA vector was used as control for determining transfection toxicity. Collagen construct expression was similar in all transfected cells as assessed by quantitative real-time PCR (qRT-PCR), on the collagen mRNA levels using a reverse primer on the engineered C-terminal tag. Differences in starting material were compensated by normalization to the endogenous reference gene L19. qRT- PCR primers are shown in Table 4.

**Table 4:** Τable 4. Primers used for quantitative real-time PCR (qRT-PCR) and ΧBP1 splicing assay.

Forward Primer		Reverse Primer	
Col4a3 RT For	CTCACGGCTGGATTTCTCTCTG	HA tag Rev	AGCGT AATCTGGAACATCGTATGGGTA
GAPDH RT For	TTGGTATCGTGGAAGGACTCA	GAPDH Rev	TGTCATCATATTTGGCAGGTTT
L19 For (XBP1 assay)	GCGGAAGGGTACAGCCAAT	L19 Rev (XBP1 assay)	GCAGCCGGCGCAAA

### Immunoblotting

Forty-eight hours after transfection cellular lysates were collected for analysis. Trans-blots were probed with a primary antibody (anti-HA, Santa Cruz Biotechnology, California, USA), targeted to the fused HA tag (*COL4A3*) epitope engineered in the carboxyl-terminus of each collagen chain. Furthermore, antibodies were used against the UPR pathway proteins such as anti-BiP, anti-p-Perk, anti-CHOP and anti-β-tubulin (Santa Cruz Biotechnology), followed by peroxidase-labelled secondary antibodies. β-tubulin was used as a loading control (Santa Cruz Biotechnology). Band density was determined using The ImageJ Software (http://imagej.hih.gov/ij). For statistical evaluation of the biochemical data we used one-way ANOVA, with Post Hoc test: Tukey’s test. For statistical significance we used the p value <0.05.

## Results

Based on our previous experience with studies on collagen IV nephropathies and also based on recent technology trends, we herewith used molecular testing as the first line of investigation in most of our patients. This was the only acceptable reasonable approach when the clinical signs and symptoms were confined to isolated GMH or GMH plus low grade proteinuria, where a renal biopsy is not normally justified. We also collected and investigated families with patients with MH of glomerular origin, some of whom had additional findings including CRF. Patients with clear-cut AS were excluded. Our basic aim was to verify how frequently patients with familial GMH had *COL4A3/A4* mutations, thereby justifying the pathological diagnosis of TBMN.

### Molecular genetics studies

All samples were first screened for the known local mutations based on previous findings [21] and if negative they were systematically screened exon-by-exon with the SURVEYOR endonuclease. In our hands the SURVEYOR endonuclease approach has 93% sensitivity while it does not exhibit any preference regarding mismatch cleavage at specific positions [23]. Variants identified in multiple samples were considered as putative polymorphisms and were further verified by testing additional control samples. When variants were present only in one or two of the 57 families under screening, they were considered as putative pathogenic mutations and familial segregation was investigated. In total, 98 nucleotide variants were identified, 8 of which are causative mutations and 90 are simple polymorphisms, 32 exonic and 58 intronic.

### Mutations identified

**Figure 1:**
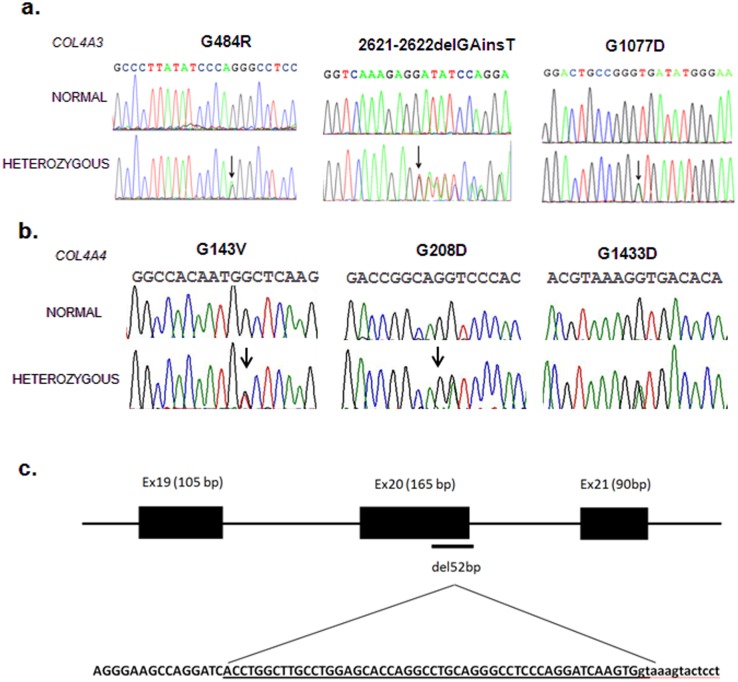
Electropherograms showing causative mutations found in *COL4A3* (a) and in *COL4A4* (b) genes during the course of this work. In (c) is shown a 52 bp deletion in *COL4A4* which encompasses 50 bp of exon 20 plus the two conserved ***gt*** bp at the donor site of intron 20. In addition to the translation frameshift introduced, it is expected that aberrant splicing will occur.

In a total of 57 consecutive families referred to our center during the period of 2009 to July 2011, 8 heterozygous mutations, four novel, were detected in 87 patients of 16 Greek-Cypriot families (28,1%). One additional novel mutation was identified in a Cypriot patient of Romanian origin. Five mutations were in the *COL4A3* and four in the *COL4A4* gene (Fig. 1). Six mutations are missense single nucleotide substitutions affecting glycines in the collagenous domain, which are known hot targets for mutagenesis. One mutation is a single guanine deletion resulting in a frameshift and a premature termination of translation, one is a 52 bp deletion and another is a combination of GA deletion/T insertion (Table 2). (summary of mutations in Table 5). Patients from two families that segregated mutations *COL4A3*-c.2621–2622delGAinsT and *COL4A3*-p.(G1077D) were married and had children who inherited both mutations and manifested classical autosomal recessive Alport Syndrome. Apart from the 52 bp deletion that was clearly pathogenic and not tested further, none of the other four novel mutations was present in any of at least 100 additional normal controls (Table 2). Also, some of these mutations tested negative on additional collections of patients with CRF or glomerulonephritis of unknown aetiology, archived in our Biobank. Some patients belonged to large pedigrees with many affected subjects based on oral medical information and biochemical testing of the recent past, but no DNA samples were available.

**Table 5:** Summary of pathogenic *COL4A3/A4* mutations found in Greek-Cypriot families studied here.

Characteristics of mutations	Number
Number of families with mutations found	16
Total number of mutations	8
Mutations in *COL4A3*/*A4*	5/3
Novel mutations	4
Mutations found in more than one family	3
Glycine substitutions (collagenous domain)	6
Small deletion	1
Insertion/deletion, frameshift	1

One additional deletion mutation was detected in a Cypriot of Romanian origin.

### Clinical characteristics

Among the 16 families we found causative mutations, we had molecular results and variable clinical information for 87 patients (38M/49F) at ages ranging from 4–78 years. Biopsies were available from patients in six families and showed TBMN and focal segmental glomerulosclerosis (Table 6). In five patients there were EM results, which confirmed the diagnosis of TBMN (Table 6). Therefore, once again molecular testing proved the best method for documenting the diagnosis. In 43 of the patients (49,4%), the heterozygous collagen IV mutation was invariably associated with isolated MH, while 12 (13,8%) had added proteinuria without CRF and another 22 (25,3%) had progressed to CRF. Importantly, 10/87 heterozygous patients (6M/4F) (11,5%) reached ESKD at ages ranging from 37-69-yo (mean 50,1-yo). Also, of 31 patients ≥51 years for whom we had clinical information, 16 patients (51,6%) developed CRF/ESKD and 5/31 (16,1%) reached ESKD. In none of the patients was there any evidence for extrarenal manifestations, such as ocular findings or hearing loss, although patients weren’t tested by specialists. It is noteworthy that in 14 of the 57 families, all 54 patients had only isolated MH or MH plus low grade proteinuria and no mutations were found, thereby implicating other unknown, perhaps less deleterious genes. These results suggest that in most families with heterozygous *COL4A3/A4* mutations and MH due to TBMN, some patients will progress to a more severe clinical outcome with age-dependent penetrance.

**Table 6:** Clinical, pathologic and mutational analysis results for the families that mutations were found in the present patient cohort.

Family	Mutationcarriers(molecularlyconfirmed)			BiopsyResults	Mutation	MH only	MH+Proteinuria(no CRF)	CRF/ESKD	Notclinicallytested	Age atESKD
	Total	♂	♀							
CY-5321	3	1	2	TBMN-FSGS(1♀)	COL4A4-c.3854delG	1♂ (64)	0	1**♀** (44)	1♂ (7)	1**♀**(37)
CY-5371	4	3	1	TBMN, FSGS (2♂)	COL4A3-p.G1334E	1♂, 1**♀** (28, 27)	0	2♂ (50, 51)	0	0
CY-5374	9	5	4	TBMN-FSGS(1♂)	COL4A3-p.G1334E	3♂, 1**♀** (62, 35, 28, 58)	1♂, 1**♀**(56, 64)	1**♀**(75)	1♂, 1**♀**(28, 30)	1**♀**(69, died at 75)
CY-5375	3	1	2	ND	COL4A3-p.G1334E	1**♀** (30)	1**♀** (57)	1♂ (64)	0	1**♂** (45)
CY-5376	11	5	6	TBMN-FSGS(1♂)	COL4A3-p.G1334E	1♂, 2**♀** (30, 4, 36)	1**♀** (36)	3♂, 2**♀** (59, 62, 38, 65, 55)	2♂ (6, 9)	2♂ (55, 37)
CY-5403	3	1	2	ND	COL4A3-p.G1334E	2♀ (31, 35)	0	1♂ (64)	0	0
CY-5352	9	2	7	ND	COL4A3-p.G1334E	2♂, 6**♀** (14, 16, 24, 26, 40, 43, 44, 55)	1**♀** (67)	0	0	0
CY-5419	12	6	6	ND	COL4A3-p.G1334E	5♂, 5**♀** (54, 28, 12, 16, 46, 53, 31, 59, 28, 30)	0	1♂ (78)	1♀ (25)	0
CY-5442	8	3	5	FSGS (1♂)	COL4A3-p.G1334E	2♀ (46, 49)	1♂, 1**♀** (53, 71)	2♂, 2**♀** (78, 45, 68, 73)	0	1**♂** (40)
CY-5346	2	0	2	TBMN-FSGS, IgM+, C3+ (1♀)	COL4A3-p.G871C	1**♀** (26)	1**♀** (49)	0	0	0
CY-5401	4	2	2	ND	COL4A3- p.G871C	2♂ (18, 36)	1**♀** (42)	1**♀** (70)	0	0
CY-5461	3	1	2	ND	COL4A3-p.G484R	0	0	1♂,1♀ (55, 57)	1♀ (34)	1♂,1♀ (55, 51)
CY-5322/4204*	2	0	2	ND	COL4A3-c.2621–2622delGAinsT	0	0	0	2♀ (63, 65)	0
CY-5322/4204*	7	7	0	TBMN-FSGS(1♂)	COL4A3-p.G1077D	3♂ (12, ?, ?)	3♂ (43, 47, 49)	1♂ (78)	0	0
CY-5430	4	0	4	ND	COL4A4-p.G143V	2♀ (53, 56)	0	1♀ (69)	1♀ (47)	1♀ (69)
CY-5324	3	1	2	ND	COL4A4-p.G208D	2♀ (44, 48)	0	1♂ (49)	0	1**♂** (43)
**SUM (%)**	87 **100**	38 **43%**	49 **57%**	8	8	18♂/25♀ 49.4%	5♂/7♀ 13.8%	13♂/9♀ 25.3%	4♂/6♀ 11.5%	6♂/4♀ **11.5%**

Of importance is a variant of unknown significance towards the C-terminal part of the collagenous domain of the α4 chain, *COL4A4*-p.(G1433D), in Cypriot family CY-5328, which was found in only one among 153 unrelated patients screened and then in six of another 120 samples of the general population. A female heterozygous carrier of p.(G1433D) developed ESKD at age 75 years, in addition to being positive for ANCA vasculitis. Her son developed ESKD at 45 years and was transplanted at 46 years. *In silico* analysis supports that it is a pathogenic mutation (Table 2). We are in the process of investigating it further with the likelihood that it may represent another founder mutation.

Finally, mutations c.2621-2622delGAinsT and p.(G1077D) in *COL4A3* were found in compound heterozygosity in members of Cypriot family CY-4204. The first mutation creates a translation frameshift and results in the addition of eight novel aminoacids after Gly-874, before it introduces a premature termination codon. Two deceased siblings had inherited both mutations and developed severe ARAS, which included sensorineural deafness and ocular lesions. One patient developed ESKD at 32 years and was transplanted twice, before he died at the age of 49 years. The truncating mutation was also identified in two heterozygous women who refused further investigation. Seven men in the same family carried mutation p.(G1077D), three of whom developed added proteinuria and one progressed to CRF.

### Next Generation Sequencing of patients who progressed to ESKD

Ten of our *COL4A3/A4* heterozygous patients progressed to ESKD (Table 6). It has been hypothesized by some that such patients might have inherited a late onset form of ARAS due to inheritance of a second *in-trans* mutation in the same gene that was missed during screening by several methods. To address this, we took advantage of the NGS technology we established in our lab towards the completion of this project. We sequenced all ten samples for all three collagen IV genes, *COL4A3/A4/A5*. In each patient we found the responsible heterozygous mutation previously detected by Sanger sequencing. Also, we found numerous synonymous and non-synonymous polymorphisms but apart from these, no additional pathogenic mutations were detected (Table 7).

**Table 7:** Synonymous and non-synonymous polymorphisms detected in *COL4A3* and *COL4A4* in this work.

Exonic changes					
	Gene	Change in DNA	Change in aminoacid	SNP code	MAF
1.	COL4A3	c.127G>C	p.G43R	rs13424243	C = 25%
2.	COL4A3	c.222G>A	p.P74P	rs187950806	A = 1%
3.	COL4A3	c.422T>C	p. L141P	rs10178458	T = 19%
4.	COL4A3	c.485A>G	p.E162G	rs 6436669	A = 19%
5.	COL4A3	c.976G>T	p.D326Y	rs55703767	T = 14%
6.	*COL4A3	c.1195C>T	p.L399L	rs10205042	C = 24%
7.	*COL4A3	c.1223G>A	p.R408H	rs34505188	A = 10%
8.	*COL4A3	c.1352A>G	p.H451R	rs11677877	G = 10%
9.	COL4A3	c.1452G>A	p.G484G	rs34019152	A = 10%
10.	COL4A3	c.1721C>T	p.P574L	rs28381984	T = 39%
11.	COL4A3	c.2501A>G	p.K834R	rs56226424	G = 2%
12.	COL4A3	c.3325C>T	p.P1109S	rs55816283	T = 1%
13.	COL4A3	c.3807C>A	p.D1269E	rs57611801	A = 4%
14.	*COL4A3	c.4665G>A	p.A1555A	rs200858199	A = 1%
15.	COL4A4	c.17T>C	p.I6T	rs16823264	C = 9%
16.	*COL4A4	c.195T>C	p.G65G	rs201278620	G = 1%
17.	COL4A4	c.1444C>T	p.P482S	rs2229814	C = 48%
18.	COL4A4	c.1634G>C	p.G545A	rs1800516	G = 3%
19.	COL4A4	c.1833T>C	p.G611G	rs145806603	G = 1%
20.	COL4A4	c.2144C>T	p.A715V	rs76636743	A = 1%
21.	COL4A4	c.2367A>T	p.G789G	rs56247709	A = 1%
22.	COL4A4	c.2899A>G	p.I967V	rs80243096	G = 2%
23.	COL4A4	c.2997G/A	p.G999G	this study	
24.	COL4A4	c.3011C>T	p. P1004L	rs1800517	C = 49%
25.	COL4A4	c.3526C/T	p.L1176L	this study	
26.	COL4A4	c.3594G>A	p.G1198G	rs10203363	A = 45%
27.	COL4A4	c.3684G>A	p.K1228K	rs2229812	A = 45%
28.	*COL4A4	c.3979G>A	p.V1327M	rs2229813	T = 45%
29.	*COL4A4	c.4080G>A	p.P1360P	rs2228556	T = 45%
30.	*COL4A4	c.4207T>C	p.S1403P	rs3752895	G = 48%
31.	*COL4A4	c.4548A>G	p.V1516V	rs2228555	T = 40%
32.	*COL4A4	c.4932C>T	p.F1644F	rs2228557	A = 46%

Intronic changes					
	Gene	Change in DNA	SNP code	MAF	
33.	COL4A3	c.144+12C>A	rs1882435	A = 35%	
34.	COL4A3	c.280–40T>G	rs55789142	G = 30%	
35.	COL4A3	c.324+73C>T	rs6750210	C = 23%	
36.	COL4A3	c.325–27C>T	rs112712399	T = 2%	
37.	*COL4A3	c.468+139C>T	rs12612699	T = 11%	
38.	*COL4A3	c.687+69G>A	rs56991885	A = 2%	
39.	COL4A3	c.687+103T>A	rs55997063	A = 7%	
40.	*COL4A3	c.765+49T>G	rs12621551	T = 19%	
41.	COL4A3	c.828+20A>G	rs13386404	G = 12%	
42.	COL4A3	c.828+59C>G	rs7559693	G = 32%	
43.	COL4A3	IVS14+85delT	this study		
44.	COL4A3	IVS14+85–86delTT	this study		
45.	COL4A3	IVS14+85 87delTTT	this study		
46.	COL4A3	c.888+30G>A	rs35467545	A = 10%	
47.	COL4A3	c.889–39T>C	rs28470865	C = 10%	
48.	COL4A3	c.933+14T>C	rs55928538	C = 1%	
49.	COL4A3	c.987+35T>G	rs10933172	G = 9%	
50.	COLA43	c.988–80T>C	rs56326869	C = 1%	
51.	COL4A3	c.1576–15T>G	rs56243460	G = 5%	
52.	*COL4A3	c.1576–60G>A	rs73993878	A = 12%	
53.	COL4A3	c.2375–66C>T	rs6436671	C = 8%	
54.	*COL4A3	c.2881+46A>G	rs6436672	A = 17%	
55.	*COL4A3	c.3419–39C>T	rs4675163	T = 11%	
56.	*COL4A3	c.3751+66C>T	rs55843236	T = 2%	
57.	COLA43	c.3883–85C>T	rs55659411	T = 4%	
58.	COLA43	IVS43–3T/A	this study		
59.	COL4A3	c.4154–69C>T	rs11680670	T = 25%	
60.	COL4A3	3′UTR+121A>G	this study		
61.	COL4A4	c.–101–4A>G	rs147186690	C = 1%	
62.	COL4A4	c.–15T>C	rs17353916	G = 1%	
63.	COL4A4	c.–2C>T	rs2228558	T = 9%	
64.	COL4A4	IVS6–56C/A	this study		
65.	COL4A4	c.490–72T>A	rs62277803	T = 2%	
66.	*COL4A4	c.490–121T>G	rs12465531	A = 38%	
67.	*COL4A4	c.595–67G>A	rs116693000	T = 1%	
68.	COL4A4	c.595–111T>C	rs6436654	T = 3%	
69.	COL4A4	c.657+48A>G	rs73082223	G = 32%	
70.	COL4A4	c.657+62G>A	rs59938187	A = 26%	
71.	COL4A4	c.657+67A>G	rs58363082	G = 26%	
72.	COL4A4	c.658–39T>C	rs12475686	T = 39%	
73.	COL4A4	c.694–72G>T	rs55741079	T = 38%	
74.	*COL4A4	c.735+104C>T	rs10174459	A = 18%	
75.	*COL4A4	c.735+202T>A	rs7600536	A = 29%	
76.	COL4A4	IVS11–67C/T	this study		
77.	COL4A4	IVS19+47G/C	this study		
78.	COL4A4	IVS21–31A/T	this study		
79.	*COL4A4	c.1029+72G>A	rs16823202	C = 41%	
80.	*COL4A4	c.2164+21T>G	rs55820723	C = 1%	
81.	*COL4A4	c.2164+78G>A	rs11898094	T = 13%	
82.	COL4A4	c.2384–5T>C	rs3769641	C = 11%	
83.	*COL4A4	c.2545+46A>T	rs7567796	T = 41%	
84.	*COL4A4	c.2545+51A>G	rs7567789	T = 38%	
85.	COL4A4	IVS34+8T/C	this study		
86.	COL4A4	c.3817+9G>C	rs13423714	G = 45%	
87.	COL4A4	c.3973+34T>C	rs1917127	C = 45%	
88.	*COL4A4	c.4081+120T>C	rs7580776	G = 7%	
89.	*COL4A4	c.4217–24C>T	rs10188770	A = 47%	
90.	*COL4A4	c.4523–8T>C	rs13419076	G = 46%	

*Polymorphisms found by NGS.

In the course of this work, 90 polymorphisms were identified, some known and some novel. Importantly 18 were non-synonymous, one of which was in the 7S domain of *COL4A3*, p.(G43R) and one in the 7S domain of *COL4A4*, p.(I6T) (Table 7).

### Cell biology-functional studies

Recently we showed that when AB8/13 undifferentiated podocytes are transiently transfected with a mutant *COL4A3* chain carrying mutation p.(G1334E), the efficiency of secretion of the mutant chain was compromised, compared to WT, as evidenced by increased intracellular retention. Also, overexpression of the mutant chain activated the UPR signalling cascade to a greater extent compared to WT chains [19]. Here we used the same approach to functionally evaluate the behaviour of several *COL4* mutant chains.

### Overexpression of wild type and mutant *COL4A3* chains in AB8/13 cells induces the unfolded protein response pathway

We used site-directed mutagenesis to introduce several mutations with the following reasoning: *COL4A3*-p.(G1334E) was previously shown to induce UPR activation [19] and served as a positive control; *COL4A3*-p.(G871C) is a founder mutation in the Cypriot population, predisposing to severe CRF/ESKD (ref. [31] and this report); *COL4A3-*p.(G484R) is close to a natural interruption of the collagenous domain; we previously hypothesised that this proximity might influence the final phenotype [31]; *COL4A3-*p.(A587G) is an artificial mutation we introduced which annuls the 10^th^ natural collagenous interruption and restores the repetitive Gly-X-Y motif.

**Figure 2:**
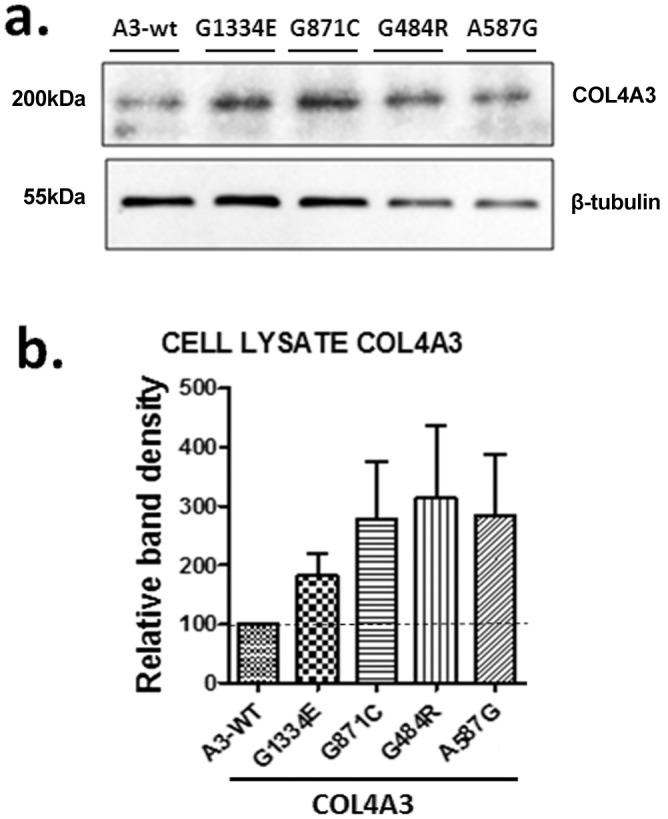
Mutant *COL4A3* chains expressed in AB8/13 cultured podocytes demonstrate a trend for increased intracellular retention. (a) AB8/13 cells were transiently transfected with expression vectors containing wild-type *COL4A3*-WT or the mutant *COL4A3* (p.G1334E, p.G871C, p.G484R, p.A587G) cDNAs that included a HA epitope at C-terminus. Single chain expression was measured via Western blot analysis of the cell lysate, 48 h after transfection. No HA antigen was detected in AB8/13 cells transfected with a construct expressing the empty vectors. Shown is a representative Western blot of proteins in cell lysates. (b) All mutant chains show a trend towards increased intracellular retention as compared to the wild type chain, although not reaching significance at the 48 h time point. Shown is densitometry analysis data normalized to tubulin expression. Data are represented as means ± SEM of n≥3 independent experiments.

Transient transfection of podocytes with equal amounts of normal single *COL4A3*-WT and mutant *COL4A3* constructs resulted in equal expression of genes as tested by qPCR (not shown). Cells expressing the mutant chains showed a trend towards higher accumulation within the cell as revealed by western blotting of the cellular lysate 48-hrs after transfection (Fig. 2). This is interpreted as the result of the ability of the cells to differentiate between normal and mutant collagen IV protomers or perhaps even single nascent polypeptide chains. It was shown previously that transfected alpha chains can be secreted either as part of triple helical molecules or as single monomeric chains. This cannot be excluded here as well [19], [32], [33].

**Figure 3:**
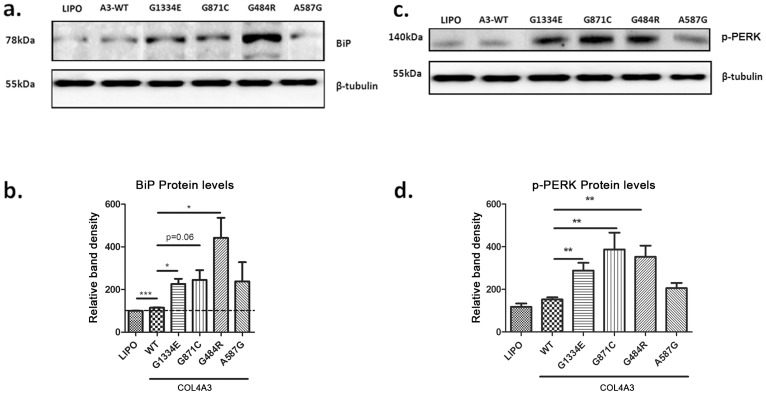
Chaperone BiP protein and PERK, a transmembrane protein kinase of the PEK family resident in the endoplasmic reticulum membrane, are deregulated in AB8/13 podocytes transfected with various *COL4A3* mutant chains. **a, c:** AB8/13 cells were transiently transfected with expression vectors containing either wild-type *COL4A3* chain or one of several mutant chains. Transfection with lipofectamine only (lipo) served as a negative control. Protein expression of the UPR markers was measured 48 hours after transfection via Western blotting. β-tubulin expression in the same samples was used as equal loading control. Shown are representative blots with differential expression levels of BiP and p-PERK for the various mutant proteins. **b, d:** Western blotting as above, was quantified via densitometric analysis. BiP and p-PERK are up-regulated in cells over-expressing the mutant *COL4A3*-p.(G1334E), *COL4A3*-p.(G871C) and *COL4A3*-p.(G484R) while there is a trend for *COL4A3*-p.(A587G), as compared to cells expressing the wild type chain. Data are means ± SEM (n = 4 for BiP; n = 7 for p-PERK) *p<0.05; **p<0.01.

We then tested for evidence of ER stress. The BiP chaperone (Grp78) is a central sensor of ER stress which provides the initial signal for UPR activation in the presence of prolonged excess or misfolding of proteins [34], [35]. Overexpression of single *COL4A3* chains resulted in a significant increase in BiP protein levels when comparing untransfected with the WT-expressing cells as well as when comparing the WT with the *COL4A3*-p.(G1334E) expressing cells, in keeping with previous results [19]. Interestingly, results also showed an increase in BiP protein between the WT and a series of other *COL4A3* mutant chain expressing cells (Fig. 3a–3b). Importantly, the same three mutations as in BiP, resulted in up-regulation of another important UPR marker, this being the activated phosphorylated PERK, p-PERK (Fig. 3c–3d) [36], [37].

### Overexpression of wild type and mutant *COL4A3* chains induces ER stress, as shown by XBP1 splicing

**Figure 4:**
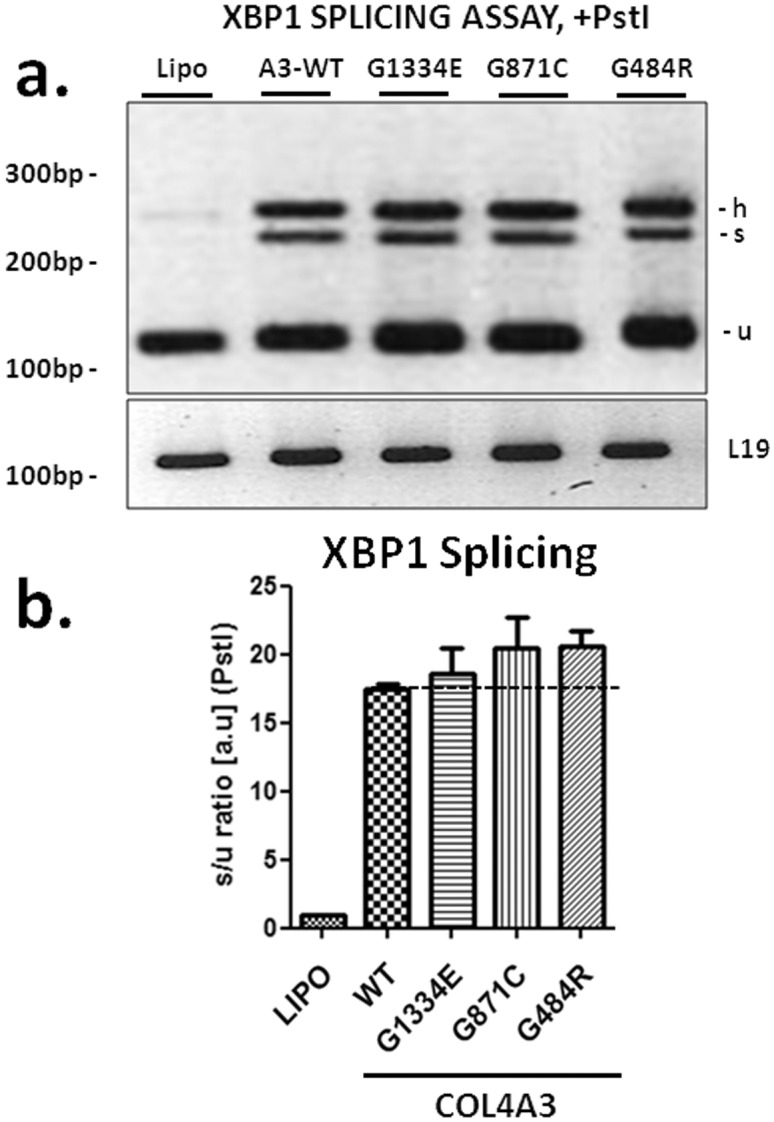
Overexpression of wild type or mutant *COL4A3* chains induces XBP1 splicing in AB8/13 cells. (a) Representative experiment of reverse transcription-PCR using XBP1 mRNA as template, from AB8/13 cells transiently expressing *COL4A3*-WT (A3/WT) or the mutant chains G1334E, G871C, G484R (*COL4A3*). PCR products were run on 3% agarose gel. It is apparent that over-expression of all chains induces XBP1 splicing, as evidenced by the appearance of the spliced band (s) when the PCR product is cut with the restriction enzyme Pst1. (h) hybrid, (u) unspliced (b) RT-PCR is quantified via densitometric analysis of the bands after PstI digestion as follows: ratio of the spliced band and the sum of the two PstI digest bands (s/(u1+u2), with PstI digestion. Hybrid band (h) was considered as equally contributing to unspliced and spliced bands. There is statistically significant XBP1 splicing when either WT or any of the mutant chains is overexpressed in AB8/13 cells. L19 was used as an internal PCR control. Data are means ± SEM of three independent experiments.

An established assay to examine UPR activation is the widely used XBP1 splicing assay [38]. Accumulation of misfolded protein within the cell’s ER causes the dissociation of BiP from IRE1 resulting in its dimerization and subsequent activation. Full length XBP1 requires the endoribonuclease domain of activated IRE1 for processing into active sXBP1, thus the splicing of XBP1 is a key marker for UPR activation. When podocytes expressed the *COL4A3*-WT chain there was obvious splicing of the XBP1 mRNA as compared to cells expressing vector-only cDNA. All mutants also demonstrated significantly more XBP1 splicing compared to cells expressing the vector-only under identical experimental conditions. Most mutants showed a trend towards higher XBP1 splicing, compared to WT, although not reaching statistical significance (Fig. 4).

## Discussion

Glomerular MH during early childhood is a common feature of a heterogeneous group of inherited hematuric nephropathies. Specifically, familial MH can be the presenting finding in patients as follows: a) In all young males and nearly all females with X-linked AS due to hemizygous or heterozygous mutations respectively, in the *COL4A5* gene. b) In all males and females who inherit ARAS due to homozygosity or compound heterozygosity for mutations in the autosomal *COL4A3/A4* genes. c) In nearly all males and females who are heterozygous for mutations in the *COL4A3/A4* genes and are diagnosed with familial MΗ due to TBMN. We say “nearly” because incomplete penetrance has been observed by several authors. d) In nearly all males and females who carry heterozygous mutations in the *CFHR5* gene, which are responsible for an inherited form of C3 glomerulopathy. A *CFHR5* exon 2–3 duplication is endemic among Greek-Cypriots. e) In males and females who inherit glomerulopathy with fibronectin deposits because they carry heterozygous mutations in the *FN1* gene for fibronectin [39].

### Genetics studies

It is currently well established that a variable but significant percentage of TBMN patients carrying heterozygous *COL4A3/A4* mutations develop later added proteinuria and CRF [1], [40]. Similar findings have been reported by several groups [7], [41], [42]. In fact some authors have data concerning extrarenal findings and ultrastructural observations that support the diagnosis of autosomal dominant AS [7], [17]. Herewith we were only interested in investigating the prevalence of heterozygous *COL4A3/A4* mutations among patients with familial MH, excluding classical AS. Our interest stemmed from previous observations that suggested a fairly high frequency of such mutations in the Greek-Cypriot population. We investigated patients from 57 families with ≥3 patients, consecutively referred to our center.

Some of our patients had progressed to proteinuria and CRF/ESKD but in none of the patients was there evidence or information for hearing loss or eye lesions. In this cohort we had biopsies from patients in 21 families (36.8%) and only in seven among those we found mutations, as this invasive procedure was not justified in the majority of cases. In all seven biopsies there was FSGS, in keeping with our previous findings which showed that FSGS can be the prevailing feature, occasionally resulting in misdiagnosis [6], [21], [43]. It is particularly interesting and revealing that among 54 patients of 14 families (average 3,85 patients per family), none had anything more than isolated MH and no mutations were detected in the *COL4A3/A4* genes. Even though the number of these families is not spectacularly high, this in its own is an indication that at least in our population, the inheritance of TBMN because of *COL4A3/A4* mutations is associated with high predisposition to proteinuria and CRF, rather than staying with isolated MH. Most probably the patients in those 14 families had inherited other unknown less deleterious genes.

We found causative mutations in 16 families, representing 28,1%. Overall, these new findings, added to our previously published data raise the total number of archived Cypriot families with familial MH to 68, with 27 of them showing heterozygous *COL4A3/A4* mutations, representing 39,7%. It was particularly rewarding that in 8 of these new 16 families studied here, we found the same previously noted founder mutation, *COL4A3*-p.(G1334E), bringing the total number of unrelated families carrying this endemic mutation to 16 with a total of 165 alive carriers. Situations like this facilitate molecular testing and diagnosis of new families. In three previous reports on smaller cohorts of 22, 21 and 40 families with MH, other researchers found *COL4A3/A4* mutations in 36%, 38% and 17,5% of families respectively [44]–[46]. In the last study, similarly to ours they had biopsy proven TBMN in only five patients.

A potential limitation of our methodology was the inability to detect easily large deletions or insertions in these genes, which may account for a subset of cases. Also, one cannot exclude the possibility that some patients may have one of the few hypomorphic mutations in the *COL4A5* gene [47]. This observation can be addressed by stating that large indels usually represent a very small percentage. Also, for base substitutions and small indels, the screening method we used by SURVEYOR digestion is of very high sensitivity in our hands [23]. At the same time though, notwithstanding all the limitations in our investigative approach, it is more than certain that additional unknown genes must exist, mutations in which may also cause familial MH or TBMN. The newly emerging NGS technology will revolutionize the speed of detection while the massive parallel analysis of tens of genes will facilitate the finding of digenic or oligogenic inheritance and the putative role of additional genetic modifiers.

### Clinical results

We had inadequate clinical data for 10/87 patients that we found mutations (Table 6). Nonetheless, important statistics that arise from this work concern the proportion of patients who demonstrated a progressive renal disease. The prognosis of these patients cannot be regarded as good if 10 of the 87 (11,5%) reached ESKD at various ages, 37-69-yo (mean 50,1-yo). Equally enlightening is the observation that among 31 patients ≥51 years that we had adequate clinical information, 16 patients (51,6%) developed CRF/ESKD and 5/31 (16,1%) reached ESKD, once again providing support against the general benign nature of TBMN due to heterozygosity for *COL4A3/A4* mutations (Table 6). This enhances the recent literature according to which MH due to TBMN can be complicated by proteinuria, FSGS and CRF on long follow-up, perhaps with the added effect of genetic modifiers [48], [49].

In several occasions the progression of heterozygous patients to CRF and even ESKD has been challenged by the suggestion that a second mutation might have been inherited *in-trans* in the same gene which accounted for the adverse outcome. Of course this cannot be the explanation for the numerous cases we and others have reported thus far, especially when there is clear familial segregation in a dominant fashion, also viewed by some as autosomal dominant AS [7], [21]. Despite this however, and because we established NGS technology towards the end of this project, we re-analysed the three *COL4A3/A4/A5* genes in 26 patients with TBMN and known heterozygous *COL4A3/A4* mutations in our Biobank, who had reached ESKD. Included were the ten patients we characterized in this report (Table 6). Notably, none of the 26 patients had a second mutation, while the NGS analysis verified all the known mutations and polymorphisms we had found in these patients by other methods. This finding adds support to the fact that even a single mutation in autosomal dominant MH, can offer the ground for a more severe progression which often times results in FSGS and/or autosomal dominant Alport-like nephritis with or without the pathognomonic ultrastructural features or extrarenal findings.

### Functional cell biology studies in a podocyte cellular model

Functional studies focused on the putative intracellular effects of WT or mutant chain overexpression on podocyte function. This project was not meant to address a thorough examination of the behaviour of every mutation. However, we wanted to generate data on at least a few more mutations for verifying our previous findings. The results demonstrated a clear trend towards increased cellular retaining of the mutant chains as compared to the WT (Fig. 2), a finding consistent with previous results [19], [32]. As regards to BiP expression, a very sensitive marker for UPR activation, as well as to p-PERK, it is interesting that there is a difference amongst the various mutants (Fig. 3). Although an *in vitro* system based on single chain overexpression is not ideal for drawing firm conclusions, still, it proved adequate to reveal the implication of the UPR pathway in the presence of collagen misfolding mutations.

The ability of *COL4* mutations involved in AS and TBMN to induce ER stress and elicit the UPR signaling is a novel finding. At cellular level, this behavior agrees with previous knowledge according to which the proper chain folding and protein quality control commence immediately after the entry of the nascent polypeptide chain in the ER, exiting the ribosomal complex. Still, we realize that the cellular system used here has limitations, mainly stemming from the single chain overexpression in podocytes and should be interpreted with caution. However, UPR induction in the presence of a *COL4A3* mutation was previously corroborated in human biopsies and in a mouse model [19].

This novel observation may in fact open a new window for therapeutic intervention in patients with collagen IV nephropathies. The observed UPR activation presumably leads to an intracellular podocyte phenotype, in addition to the GBM defects resulting from impaired mature protomer secretion. One hopes that modulation of the UPR cascade with external synthetic chaperones might alleviate the negative features by promoting secretion of partly functional mutant collagen trimers. It may turn out that partially functional collagen IV molecules in the GBM network are better than null secretion [50]. Preliminary experiments in cultured podocytes showed that external administration of synthetic chaperones promotes increased secretion rather than retention of mutant chains (Papazachariou L, Pieri M, Deltas C; unpublished results).

It would be premature to draw firm conclusions; however the difference observed in BiP and p-PERK expression in the cells, elicited by expression of different mutants, reveals that the cells have a way of differentiating the handling of abnormal chains, something that may or may not have significance in the final phenotype. The newly introduced *COL4A3-*p.(A587G) for restoring the Gly-X-Y motif, although not reaching statistical significance, showed evidence for BiP and p-PERK induction, thus supporting the notion that naturally existing interruptions of the collagenous domain exert important functions. The overall differential handling of the various mutations by the cell is a strong indication of its ability to sense biochemical differences emanating from different aminoacid substitutions. Consequently, this might be considered in future experiments for functional evaluation of *COL4* mutations that could be extended to the final patient prognosis. A note of caution regards the fate and destination of the singly transfected collagen chains. We showed before that the AB8/13 cells used here, do express endogenously the collagen IV α3-α5 chains [19]. It is reasonable therefore that part of the overexpressed chains associate with endogenous to form mature trimers; consequently, any defective misfolded trimers activate the UPR pathway. It is also probable though that some single chains might be secreted, perhaps due to the overexpression, thereby escaping the system checkpoints which require that only trimers are secreted [33], [51], [52].

## Conclusion

In conclusion, our findings substantiate the observation that familial MH in about 40% of cases is caused by heterozygous *COL4A3/A4* mutations, while it is more than certain that additional still unknown genes exist that are responsible for a similar phenotype. As the DNA analysis technology is improving and NGS approaches are becoming routine practice, more experts may prefer to use genetic testing as a first-tier approach for diagnosis, before they resolve to an invasive method such as a skin or a renal biopsy [53]. The several approaches and their *pros* and *cons* were discussed during the recent Alport Workshop in Oxford, England (January 2–5, 2014) [54].

At the same time our data enhance the notion that these heterozygous mutations predispose highly to FSGS and CRF/ESKD usually at advanced age, a phenotype that is reminiscent to autosomal dominant Alport-like nephritis, with or without the classical AS features. In fact, TBMN associated with heterozygous *COL4A3/A4* mutations and FSGS histology, emerges as a more frequent cause of ESKD than classical AS. Also, our functional studies in cultured podocytes showed that overexpression of WT or mutant COL4 chains causes increased retaining of mutant chains in cell lysates. Even more important was the observation that some mutations differentially activate the UPR pathway, thereby contributing to an intracellular phenotype which is expected to complicate the phenotype because of the defective GBM.
